# Real-World Data of R-mini-CHOP Therapy in Elderly Hispanic Population with Diffuse Large B-Cell Lymphoma and High-Grade Follicular Lymphoma

**DOI:** 10.3390/cancers18071124

**Published:** 2026-03-31

**Authors:** Carla Romagnoli, Veronica Guerra, Leily Santos-Carrion, Marisol Ocampo, Alexandra Lyubimova, Evelyn Goya Balaguer, Yelida Brauchle, Oleg Gligich, Bruno Bastos, Aron Simkins, Arnold Blaustein, Michael Schwartz, Mike Cusnir, Jacqueline C. Barrientos

**Affiliations:** 1 Division of Hematology/Oncology, Mount Sinai Medical Center, Miami Beach, FL 33140, USAoleg.gligich@msmc.com (O.G.);; 2 Department of Hematology, Washington University School of Medicine, St. Louis, MO 63310, USA

**Keywords:** R-mini-CHOP, diffuse large B-cell lymphoma, elderly, Hispanics

## Abstract

**Background/Objectives**: Diffuse large B-cell lymphoma (DLBCL) and high-grade follicular lymphoma (FL) are aggressive B-cell malignancies predominantly affecting older adults. R-CHOP remains the frontline standard of care, with frail and elderly patients requiring attenuated regimens such as R-mini-CHOP. Real-world comparative data in elderly and Hispanic populations remain limited. We aimed to evaluate outcomes of R-mini-CHOP versus R-CHOP in elderly patients and to explore potential differences by ethnicity. **Methods**: Single-center retrospective analysis of adult patients older than 70 years with DLBCL and high-grade FL, treated between January 2014 and June 2025. Clinical characteristics, treatment responses, and survival outcomes were analyzed. The overall survival (OS) and progression-free survival (PFS) were estimated using the Kaplan–Meier method. **Results**: A total of 136 patients were included (72 R-mini-CHOP; 64 R-CHOP). Patients receiving R-mini-CHOP were older (median 82 vs. 74 years) and had higher-risk features. Overall response rates were 88.7% and 92.6% in the R-mini-CHOP and R-CHOP groups, respectively. Two-year OS was 79.3% for R-mini-CHOP and 76.7% for R-CHOP. Median OS and PFS were not reached in either group. Elevated lactate dehydrogenase (LDH) was associated with an inferior response. We identified a trend toward better response with R-CHOP in Hispanic patients, although this was not statistically significant. **Conclusions**: In this real-world cohort, R-mini-CHOP achieved response and survival outcomes comparable to R-CHOP despite worse baseline characteristics. These findings support the use of dose-attenuated therapy in frail and elderly patients and suggest that equitable access to care may mitigate ethnic disparities in outcomes.

## Simple Summary

Older adults with aggressive B-cell lymphomas are often treated with intensive chemotherapy. However, frail and very elderly patients frequently require reduced-dose regimens, like R-mini-CHOP. Real-world data in this setting, particularly in Hispanic populations, remains limited. In this study, we evaluated outcomes of elderly patients treated at a single cancer center serving a large Hispanic community. We compared reduced-intensity therapy with standard treatment and examined whether ethnicity influenced survival. We found that patients receiving the reduced regimen achieved response rates and two-year survival similar to those of patients receiving standard therapy despite being older and having more high-risk disease features. We also did not observe differences in survival by ethnicity in this setting. These findings support the use of dose-adjusted therapy in frail and elderly patients and highlight the importance of equitable access to specialized cancer care.

## 1. Introduction

Diffuse large B-cell lymphoma (DLBCL) is the most common lymphoma subtype, with a median age at diagnosis of 67 years [1]. The Surveillance, Epidemiology, and End Results (SEER) data revealed racial and ethnic disparities, with the highest incidence among non-Hispanic White people followed by Hispanic people. These trends have remained stable in recent years. In addition to de novo DLBCL, high-grade follicular lymphomas (grade 3B) represent an important subset of aggressive B-cell lymphomas with similar clinical behavior and treatment strategies [2,3].

Standard treatment over the past two decades for both entities has consisted of rituximab, cyclophosphamide, doxorubicin, vincristine, and prednisolone (R-CHOP). Although fit elderly patients can benefit from standard chemoimmunotherapy regimens, most frail or unfit patients are unable to tolerate the full dose of the regimen due to myelosuppression and other complications. These patients are usually started on reduced-dose regimens, such as R-mini-CHOP [4]. This regimen includes a 50% reduction in the total dosages of anthracycline and cyclophosphamide, along with adjustments to the dosages of vincristine and prednisone. Alternative regimens have been explored in this population but are generally associated with poor long-term survival, including R-CVP, which excludes doxorubicin, as well as rituximab plus bendamustine or lenalidomide [5]. Some of these approaches, especially non-anthracycline-containing regimens, should be regarded as palliative rather than curative.

Reported 2-year overall survival (OS) of patients 80 years and older treated with R-mini-CHOP ranges from 59 to 68% [1,6]. Age and disease stage are widely known key factors that affect outcomes of cancer patients, but little is known about outcomes in elderly and frail patients based on their race and ethnicity. According to the latest SEER population-based statistics, non-Hispanic Black patients diagnosed with aggressive non-Hodgkin lymphoma continue to be among those with the poorest prognosis, showing similar outcomes for OS among Hispanic and non-Hispanic patients. Data on survival outcomes for Hispanic patients with aggressive lymphomas in different publications show similar OS among non-Hispanic patients; however, this population has been historically underrepresented in all studies. Racial and ethnic minority patients with DLBCL were demonstrated in previous studies to have a more advanced disease and experience inferior survival outcomes compared with White patients [7].

This study aimed to determine the outcomes in elderly populations diagnosed with DLBCL and high-grade follicular lymphomas treated with R-mini-CHOP and R-CHOP at a single institution serving a large community of Hispanics and to explore any disparities in survival outcomes between racial and ethnic groups.

## 2. Materials and Methods

### 2.1. Study Population

A retrospective cohort study was conducted at the Mount Sinai Cancer Center in Florida, including patients aged 70 years and older who were diagnosed with DLBCL or high-grade follicular lymphoma, following the World Health Organization (WHO) classification of lymphoid neoplasms.

The patients included in this study were diagnosed between January 2014 and June 2025 and received at least 1 cycle of either R-CHOP or R-mini-CHOP. Those receiving palliative therapy or with incomplete data were excluded. Patients who had R-CHOP doses adjusted to less than 80% of the original dose for a single drug in the regimen were classified within the R-mini-CHOP group.

Treatment selection was not based solely on chronological age but rather on physician clinical judgment, incorporating factors such as performance status, comorbidities, frailty, and overall fitness, in addition to age.

### 2.2. Study Variables

This study collected comprehensive data including demographic variables like age and self-reported sex, race, and ethnicity; clinical characteristics like Eastern Cooperative Oncology Group (ECOG) performance status, Charlson comorbidity index, diagnosis and cell of origin, Ann Arbor stage, extranodal disease, bone marrow disease, international prognostic index (IPI) and CNS-IPI scores, transformation status, and molecular markers (BCL2, BCL6, EBER, MYC, lactate dehydrogenase [LDH], and beta 2 microglobulin). Double-HIT lymphoma was defined as the presence of concurrent MYC and BCL2 and/or BCL6 rearrangements detected by fluorescence in situ hybridization (FISH). In contrast, triple-HIT lymphoma included rearrangements in all three genes. Patients were identified through a systematic query of the institutional electronic medical records (EMRs) database following approval by the local Institutional Review Board (IRB). This study was conducted in accordance with the Declaration of Helsinki, and, given its retrospective nature, a waiver of informed consent was granted. Eligible cases were confirmed by pathology reports and clinical documentation to ensure diagnostic accuracy. Outcome variables included overall response rate (ORR), OS, progression-free survival (PFS), and event-free survival (EFS).

### 2.3. Statistical Analysis

Baseline characteristics were evaluated using chi-square or Fisher’s exact test for categorical variables and the Mann–Whitney U test for continuous variables. Predictors of complete response (CR) were assessed using univariate and multivariate logistic regression.

Overall survival was analyzed using the Kaplan–Meier method and Cox proportional hazards regression. The proportional hazards assumption was evaluated using time-dependent covariates. NCCN-IPI violated this assumption and was therefore included as a stratification factor in the final Cox regression model. Due to the limited number of outcome events, multivariate models were restricted to a small number of clinically relevant covariates. A two-sided *p*-value < 0.05 was considered statistically significant. Analysis was performed using SPSS version 31.0.0.0 and GraphPad Prism 10.

We performed an exploratory sensitivity analysis of the Hispanic subgroup using univariate Cox regression to assess whether the associations were consistent with those in the overall cohort. Multivariate analysis was not performed due to the limited number of events.

## 3. Results

### 3.1. Population Characteristics

A total of 136 elderly patients aged 70 years and older were included; 72 patients received R-mini-CHOP and 64 received R-CHOP. We observed a tendency toward increased use of R-mini-CHOP, particularly among patients over 80 years old. Notably, most R-mini-CHOP treatments (67%) were initiated after 2020, suggesting a temporal shift in clinical practice toward greater adoption of the attenuated regimen in this population.

- R-mini-CHOP cohort: The median age was 82 years (median range 79 to 85), 58% (*n* = 42) were female, 80.5% had ECOG ≤ 1, and 58% were Hispanic. Most patients were diagnosed with DLBCL (86%), the majority non-germinal center type (63%). Risk stratification showed that 73.6% had III-IV Ann Arbor stage disease, 30.6% had a high-risk IPI score, and 30.6% of them had an NCCN-IPI high-risk score. Elevated LDH levels were observed in 31 of 72 patients (43%), 18 of whom were Hispanic (58%). Among molecular characteristics, 35 patients were identified as double expressors, 3 as having a double-HIT lymphoma, and 1 as having triple-HIT lymphoma (Table 1). Sixteen of seventy-two patients (22%) died during follow-up, of whom disease progression was confirmed in four cases.
- R-CHOP: The median age was 74 years (median range 71 to 77), 55% (*n* = 35) were female, all had ECOG ≤ 1, and 48.4% were Hispanic. DLBCL was the predominant diagnosis (87.5%). Risk stratification showed that 62.5% had III-IV Ann Arbor stage disease, 15.6% had high-risk IPI, and 6% had a high-risk NCCN-IPI score. Elevated LDH levels were observed in 29 of 64 patients (45%), 12 of whom were Hispanic (41%). The prevalence of double expressors was 33%, and there were no patients with double-HIT lymphomas and just one with triple-HIT lymphoma (Table 1).

### 3.2. Treatment Response

**Figure 1:**
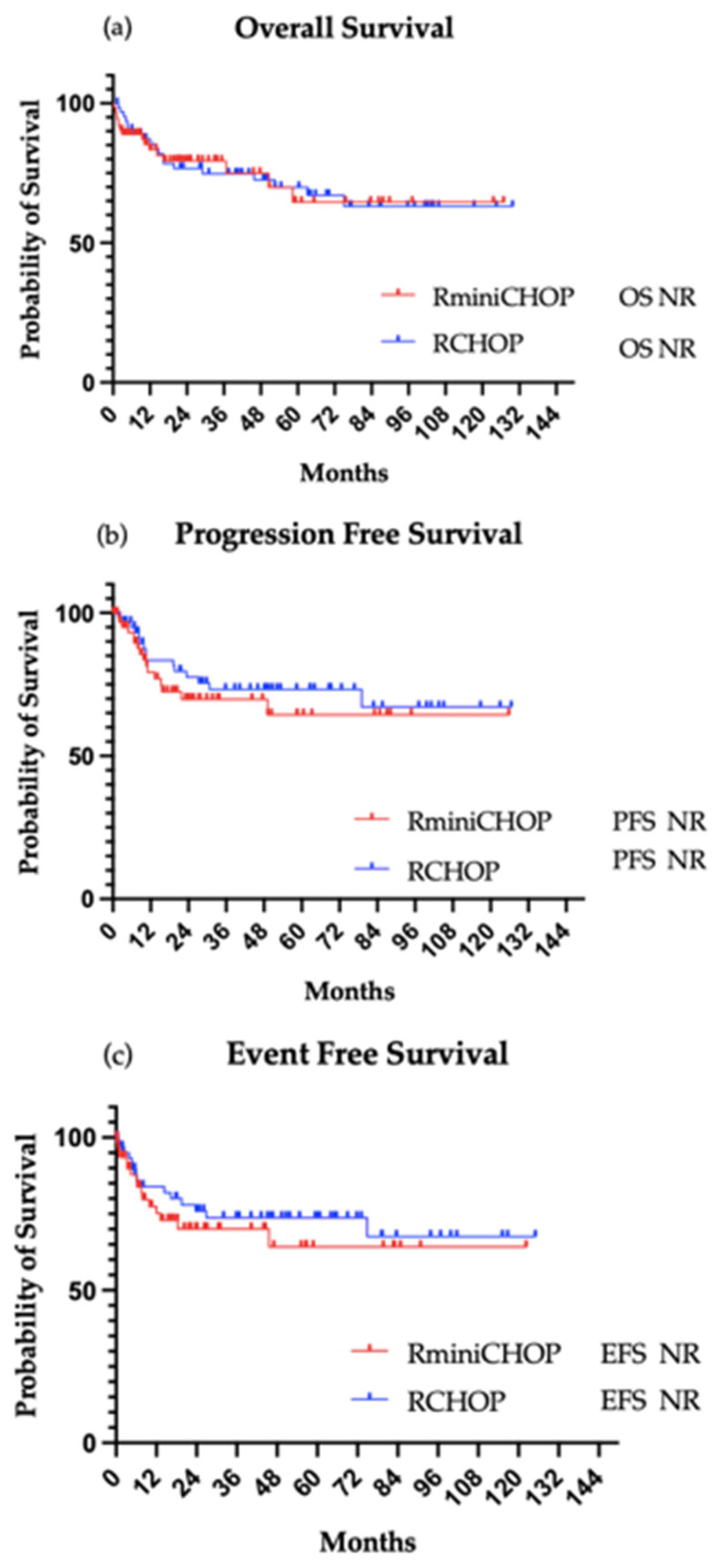
Survival curves. (**a**) Overall survival (HR 1.010; 95% CI 0.52 to 1.96, *p* = 0.976); (**b**) progression-free survival (HR 1.307; 95% CI 0.64 to 2.65, *p* = 0.453); (**c**) event-free survival (HR 1.303; 95% CI 0.64 to 2.64, *p* = 0.457).

In the R-mini-CHOP group, 82% of patients (*n* = 59) completed 3 or more cycles of therapy, with a median of 6 cycles (range 1 to 8). Among 53 evaluable patients, 44 achieved CR, 3 had partial responses (PR), and 6 progressed. With a median follow-up of 24.6 months, the ORR was 88.7%, and the 2-year OS was 79.3% (Figure 1).

Among R-CHOP-treated patients, 54 patients were evaluable for response. With a median follow-up of 64.2 months, the ORR was 92.6%, and the 2-year OS was 76.7%. Median OS, PFS, and EFS were not reached in either group. In the Cox regression model stratified by NCCN-IPI risk group, after adjusting for sex, treatment regimen was not significantly associated with overall survival (HR 0.79, 95% CI 0.38–1.61, *p* = 0.516) (Appendix A Table A1).

### 3.3. Comparison Between Treatment Groups and Predictors of Response

**Figure 2:**
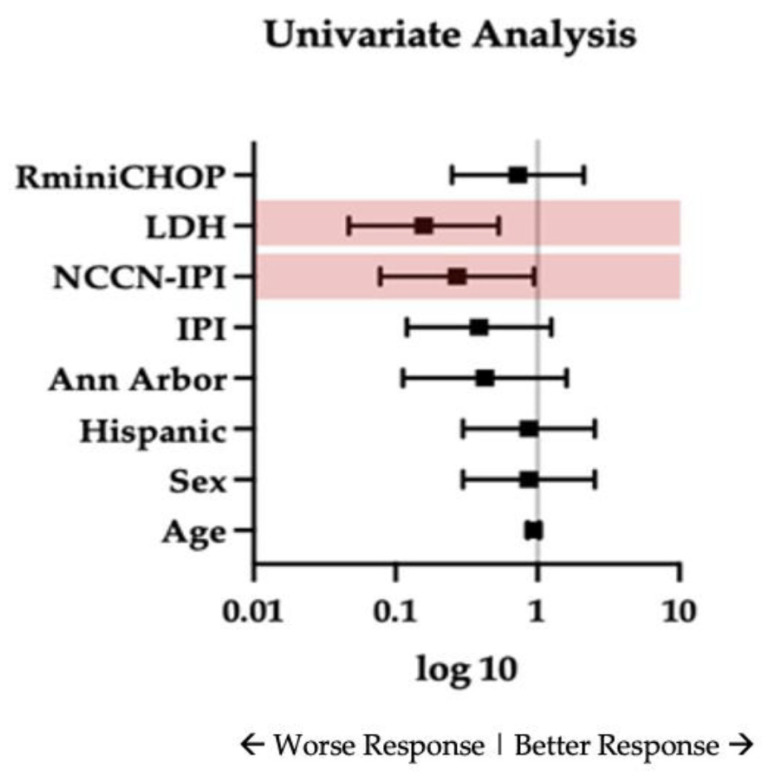
Predictors of response. Univariate analysis of predictors of good response. Forest plot displaying odds ratios (ORs) with 95% confidence intervals for baseline clinical characteristics and treatment variables. Outcome is defined as achieving a good response. OR < 1 reflects a lower likelihood of response, and OR > 1 indicates an increased likelihood of response. The vertical line corresponds to OR = 1 (no association). Arrows indicate the direction of response, with values to the left representing worse response and values to the right representing better response.

Response rates were compared within the two groups. Age ≥ 80 years and high-risk IPI or NCCN-IPI were significantly associated with treatment allocation (*p* ≤ 0.0001, 0.0451, and 0.0003, respectively). Univariate analysis identified elevated LDH as a predictor of inferior response (OR 0.158; 95% CI 0.05–0.53; *p* = 0.003) and high-risk NCCN-IPI (OR 0.272; 95% CI 0.08–0.94; *p* = 0.04) (Figure 2, Appendix A Table A2). After adjusting for treatment, high-risk NCCN-IPI was associated with lower odds of achieving response (OR 0.28, 95% CI 0.07 to 1.02) compared with low and intermediate-risk NCCN-IPI. However, this difference was not statistically significant (*p* = 0.053).

**Figure 3:**
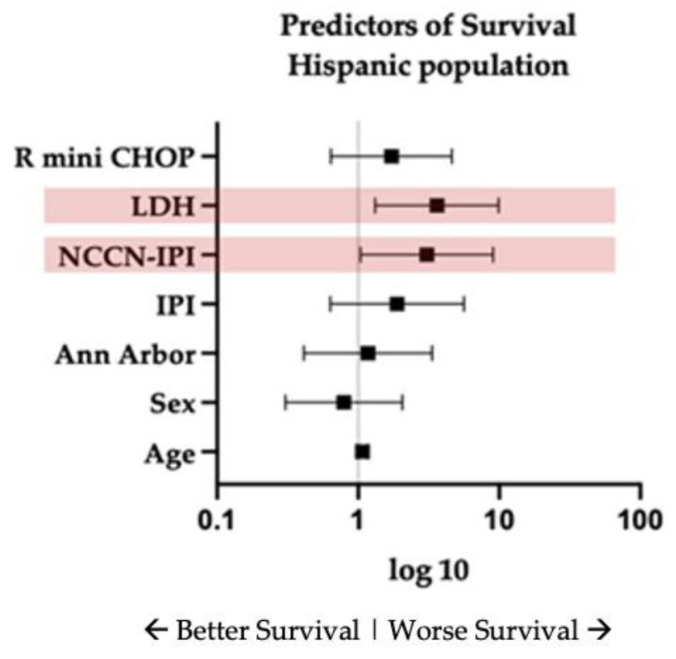
Predictors of survival among the Hispanic treated population. Forest plot displays hazard ratios (HRs) with 95% confidence intervals for clinical and treatment-related variables. HR > 1 indicates an increased risk of death, HR < 1 indicates a reduced risk of death. The vertical line corresponds to HR = 1 (no association). Arrows indicate the direction of survival, with values to the left representing better survival and values to the right representing worse survival outcomes.

**Figure 4:**
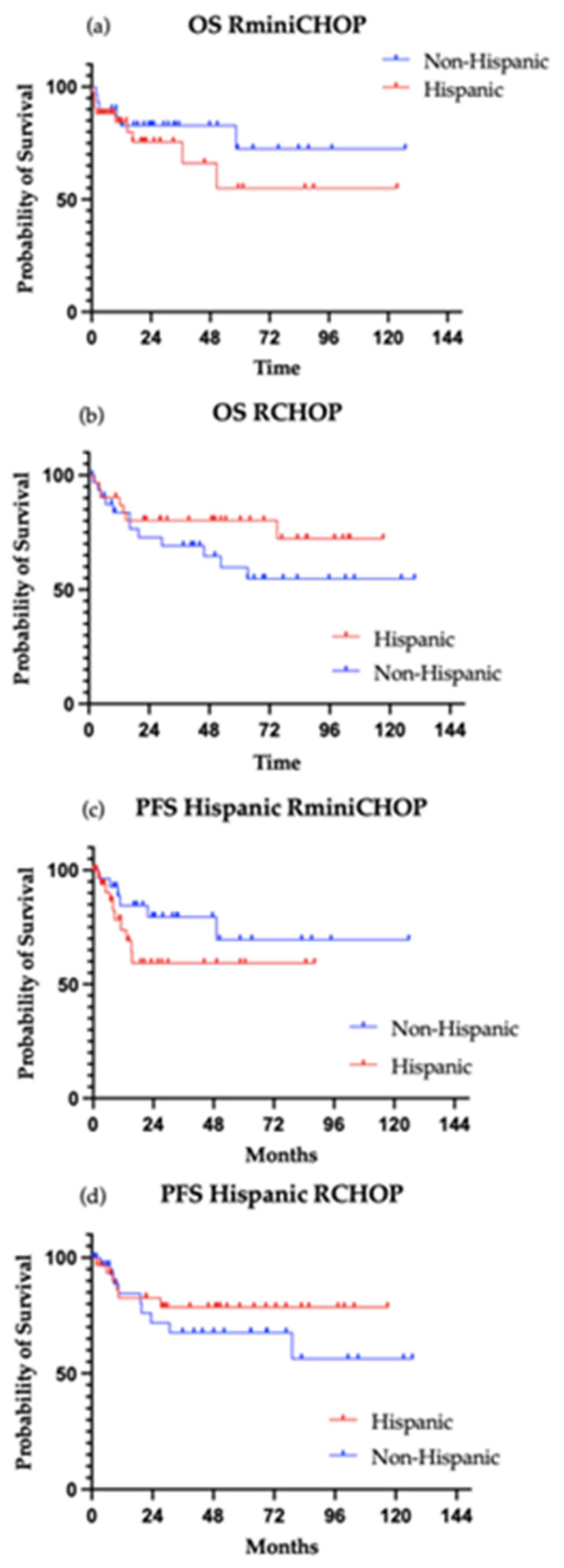
Survival outcomes of Hispanic patients. (**a**) Overall survival in Hispanic patients treated with R-mini-CHOP (HR 1.626; 95% CI 0.61 to 4.33, *p* = 0.3379); (**b**) overall survival in Hispanic patients treated with R-CHOP (HR 0.5451; 95% CI 0.22 to 1.34, *p* = 0.1946); (**c**) progression-free survival in Hispanic patients treated with R-mini-CHOP (HR 1.938; 95% CI 0.72 to 5.18, *p* = 0.1895); (**d**) progression-free survival in Hispanic patients treated with R-CHOP (HR 0.6049; 95% CI 0.22 to 1.67, *p* = 0.3340).

In the exploratory sub-analysis of Hispanic patients, the direction and magnitude of the associations were consistent with those in the overall cohort. High-risk NCCN-IPI and elevated LDH were associated with inferior overall survival in univariate analysis, while treatment regimen was not significantly associated with survival (Figure 3, Appendix A Table A3). Overall survival and PFS for Hispanic patients were not reached (Figure 4).

### 3.4. Exploratory Subgroup Analysis

A subgroup analysis was conducted on patients treated with the current therapeutic approach, Pola-R-mini-CHP (as per the POLAR BEAR trial in progress). However, this subgroup was not included in the overall analysis due to the relatively recent adoption of the regimen and the limited number of patients treated with it. As a result, the differences observed did not reach statistical significance and are only descriptive.

## 4. Discussion

This single-center analysis represents one of the largest real-world cohorts of elderly Hispanic patients with aggressive B-cell lymphomas treated with curative intent. Prior studies from Latin America provided important insights into the clinical characteristics, treatment patterns, and outcomes of elderly Hispanic patients [8,9]. However, these reports primarily included heterogeneous treatment strategies across multiple institutions and were not designed to specifically characterize outcomes of curative-intent therapy within a single Hispanic cohort. Another important study conducted in New York examined racial and ethnic differences in survival among patients with DLBCL, reporting superior survival outcomes among Hispanic/Latino patients compared with non-Hispanic White patients. However, the analysis did not provide detailed information on treatment regimens or curative-intent therapies and primarily focused on survival disparities within an underserved population [10].

According to the most recent official U.S. Census data from 2020, 26.5% of Florida’s population was of Hispanic origin, and projections suggest that Hispanics will remain the largest ethnic and racial minority in the state. Furthermore, epidemiological projections indicate that the incidence of DLBCL is expected to increase in the coming decades, in contrast to relatively stable rates of FL, largely driven by the growing population aged ≥65 years [6,11]. Given the projected increase in DLBCL incidence among older adults, understanding outcomes with attenuated regimens in this population is critical.

Population-based data underscore that chronological age alone should not guide treatment decisions. Lowsky et al. showed that even among the oldest individuals, nearly one third had not been diagnosed with any of the five major chronic diseases (cancer, diabetes, heart disease, lung disease, and stroke), and those aged ≥85 years were 58% as likely to require help with activities of daily living compared with those aged 51–54 [12].

In the lymphoma-specific setting, a recent meta-analysis demonstrated that comprehensive geriatric assessment (CGA) before treatment initiation was associated with significantly improved OS in older adults with DLBCL [13]. In this context, Li et al. conducted a meta-analysis evaluating the association between malnutrition and clinical outcomes. Malnutrition, assessed using multiple screening tools, was consistently associated with worse survival outcomes, underscoring the prognostic relevance of baseline nutritional status, particularly in elderly and vulnerable populations [14].

Furthermore, in the REFLECT study evaluating health-related quality of life in 169 patients with DLBCL, better baseline cognitive, physical, or role functioning, as well as fewer symptoms such as appetite loss, diarrhea, fatigue, or pain, were associated with a higher likelihood of achieving a complete rather than partial response at the end of treatment [15]. Overall, these findings highlight a critical point often overlooked: functional status and comorbidity assessment can be just as, if not more, important than chronological age in guiding therapeutic decisions [16,17].

Notably, in our cohort, Hispanic ethnicity was equally distributed between the young and fit versus the older and frail groups. R-mini-CHOP was preferentially used in patients with advanced Ann Arbor stages and high-risk scores (IPI ≥ 4 and NCCN-IPI ≥ 6). The differences between the two groups, high-risk IPI and NCCN-IPI, were statistically significant (*p* = 0.0451 and *p* = 0.0003), as was age over 80 years (*p* ≤ 0.0001). Additionally, there was a modest increase in the prevalence of double expressors and in double- and triple-HIT lymphomas within the R-mini-CHOP group. However, these differences did not reach statistical significance. This is consistent with other studies showing that fit patients benefit from full-intensity curative therapy, while intermediate-dose regimens do not significantly compromise outcomes in frail patients [18]. A multicenter study showed that higher dose intensities were associated with increased toxicity and worse outcomes; however, when relative dose intensity fell below 70%, relapse rates increased markedly, highlighting the narrow therapeutic window in this population [19].

To date, only one meta-analysis has compared the efficacy and safety of different first-line treatments in this population. This meta-analysis included 1839 patients and demonstrated a PFS advantage for patients treated with the R2CHOP-21 (lenalidomide plus R-CHOP administered every 21 days) compared with R-COMP (R-CHOP without doxorubicin), R-miniCEOP (with epirubicin replacing doxorubicin), RCHOP-14 (administered every 14 days), and RCHOP-21. No statistically significant differences in ORR, CR, and OS were demonstrated between these regimens [20]. Notably, R-mini-CHOP was not included in this network meta-analysis because it has not been evaluated in randomized comparative trials and is primarily supported by single-arm phase II studies in very elderly (≥80 years) and frail patients, precluding reliable indirect comparisons based on hazard ratios.

Several reports have demonstrated that the relative incidence of activated B-cell (ABC) vs. germinal center B-cell (GCB) subtypes significantly increases with age and is associated with higher prognostic risk [6,21]. Despite adverse baseline features, ORR was similar between both groups (88.7% for R-mini-CHOP vs. 92.6% for R-CHOP), and 2-year OS was nearly identical (79.3% vs. 76.7%, respectively), supporting the regimen’s efficacy and tolerability in the very elderly. Elevated LDH levels predicted worse outcomes, including in the Hispanic subgroup. Although the overall proportion of patients with elevated LDH was comparable between treatment groups (43% in R-mini-CHOP vs. 45% in R-CHOP), Hispanic patients were more frequently represented among those with elevated LDH in the R-mini-CHOP cohort (58% vs. 41%). This finding suggests that Hispanic patients with higher disease burden may have been preferentially treated with reduced-intensity therapy, which could potentially influence outcomes. Similarly, in a real-world evidence study in a Brazilian population, high LDH levels were identified as an independent prognostic factor associated with poor outcomes [22].

These findings contrast with results from prior studies, such as the LNH03-7B trial performed by Groupe d’Etude Des Lymphomes de l’Adulte (GELA) reported in 2010, which included 150 patients [23]. In that study, an ORR of 74% was observed, with a median follow-up of 20 months, a 2-year OS of 58.9% (95% CI, 49.3–67.2%), and a 2-year PFS of 47.4% (95% CI, 38.1–56.2%).

Our results are consistent with prior prospective evidence, such as the phase 2 trial presented by Peyrade et al., which evaluated the R-mini-CHOP regimen in patients older than 80 years. They demonstrated the safety and feasibility of this approach, reporting an ORR of 73%, a 2-year OS of 59% (49–67%), and a 2-year PFS of 47% (38–56) [24]. As summarized by Kumar et al., the study established R-mini-CHOP as a balanced and reasonable treatment option for selected octogenarians who are unlikely to tolerate a full-dose regimen, offering a good balance between clinical outcome, efficacy, and safety profile [25].

Some population-based analyses, however, have reported better survival with full-intensity chemotherapy. A study by Al-Sarayfi et al. reported a 2-year OS for patients treated with R-mini-CHOP that was significantly lower than that for R-CHOP (60% vs. 75%, *p* < 0.01) [26]. A systematic review evaluating the impact of R-CHOP dose intensity on survival outcomes demonstrated that reduced dose intensity was associated with inferior outcomes. However, among patients aged ≥ 80 years, the effect of dose intensity on survival was not consistent, suggesting a nuanced risk–benefit balance in this very elderly population [27].

In a secondary analysis of the phase III CALGB 50303 trial (Alliance 151930), age-related toxicity and outcomes were evaluated among patients with newly diagnosed DLBCL treated with R-CHOP. Although older patients (≥65 years) were able to complete standard therapy at rates comparable to younger patients, they experienced significantly higher rates of grade ≥ 3 non-hematologic toxicities. In contrast, severe hematologic toxicity did not differ by age. Importantly, although age had no impact on ORR, OS was inferior in patients ≥ 65 years after adjusting for disease stage, extranodal disease, and ECOG performance status. In contrast, 1-year and 3-year PFS were not impacted by age group after adjusting these variables, likely reflecting increased treatment-related complications and non-lymphoma-related mortality. These findings highlight the limitations of standard-dose R-CHOP in older adults and support the development and use of attenuated regimens, such as R-mini-CHOP, to improve tolerability while maintaining disease control in elderly patients [28].

Taken together, these data underscore that chronological age alone often misrepresents an individual patient’s capacity to tolerate therapy. In our experience, frailty, comorbidity burden, and social support play equally critical roles, which may explain why some octogenarians tolerate full-dose R-CHOP remarkably well despite advanced age. We also believe in the importance of timely care support interventions, such as myeloid growth factors, primary antimicrobial prophylaxis, and pre-phase therapy, to optimize outcomes in this population. Additionally, older patients with lymphoma face an increased risk of low bone mineral density, which may be exacerbated by treatment, suggesting that high-risk patients should be considered for bone protection strategies also [29].

However, this study has several important limitations. First, our analysis was restricted to patients who received cancer-directed therapy; therefore, we were unable to characterize outcomes among untreated patients or to fully assess potential differences in treatment selection and outcomes across age groups. Second, treatment allocation was not randomized, and residual confounding may have influenced the observed outcomes. In particular, patients who received R-mini-CHOP were older and may have had a higher burden of comorbidities or frailty, which could impact both treatment selection and outcomes. Finally, although our cohort represents a racially and ethnically diverse population, our results are based on a single institution, which may limit the generalizability of the findings. Replication of this study in larger, multi-institutional cohorts will be necessary to better evaluate the real-world use and outcomes of this chemotherapy regimen across broader populations.

### Emerging Therapies and Future Implications

Many efforts are ongoing to improve standard frontline treatment. Recently, the phase 3 POLARIX study demonstrated that treatment with polatuzumab vedotin-R-CHP had a significant but small PFS benefit over R-CHOP, though complete remission rates did not improve and there was no benefit in OS [30]. The 5-year update showed sustained PFS advantage, with a 5-year PFS rate of 64.9% (95% CI, 59.8–70) for Pola-R-CHP and 59.1% (95% CI, 53.9–64.3) for R-CHOP, but similar OS, with exploratory analyses suggesting benefit with Pola-R-CHP in high-risk subgroups, including ABC subtype and IPI scores of 3–5 [31]. Post hoc analyses in patients ≥ 60 years indicated a clinically meaningful improvement in PFS, particularly for those ≥70 years (HR 0.63, 95% CI 0.41–0.96), while OS remained similar [32]. Beyond age considerations, real-world data also suggest promising activity of Pola-R-CHP in biologically high-risk subgroups such as dual-expressor DLBCL, including elderly patients, with overall response rates exceeding 95%, although a higher rate of early treatment failure in older individuals was seen [33].

In the real-world setting, a recent study evaluated the association between age, fitness, and dose intensity with the safety and efficacy of first-line pola-RCHP in older adults with DLBCL. A dose-adjusted regimen (pola-R-miniCHP) was administered to patients aged 80 years or older. Comparable response rates and survival outcomes were observed between older and younger patients, and dose attenuation in the elderly population did not appear to compromise efficacy [34]. These findings were further supported by a single-institution Japanese retrospective study including patients ≥ 80 years treated with pola-R-miniCHP, which reported a 100% complete response rate among evaluable patients and a cumulative survival rate of 94.7% at a median follow-up of 8.9 months, with manageable toxicity and low non-relapse mortality [35]. In addition, a broader real-world analysis of frail patients aged ≥60 years or with significant comorbidities treated with polatuzumab-based regimens demonstrated an ORR of 90.7% and CR of 53.7%, with acceptable grade 3–5 toxicities and encouraging early survival outcomes [36].

Although exploratory, our preliminary data on outcomes with Pola-R-mini-CHP align with these published results, suggesting this combination may become a future standard of care for elderly, unfit patients. Continued follow-up and expanded cohorts are necessary to draw reliable conclusions, which should be reported in future analyses. It is essential to note that although new targeted agents are being developed, they are currently only available in clinical trials. For many elderly patients, participation is challenging due to the strict eligibility criteria.

Interestingly, while disparities in health outcomes due to race and ethnicity have been consistently reported in the literature, we did not observe comparable differences in our cohort. This may be explained by the characteristics of our population, which largely comprises Hispanic patients with reliable access to comprehensive cancer care. This population is predominantly characterized by a relatively high educational level and consistent access to specialized oncologic services, factors that have been shown to mitigate outcome disparities in other hematologic malignancies, such as chronic lymphocytic leukemia [37]. Consequently, our results may not be generalizable to Hispanic patients who lack access to tertiary care centers, specialized multidisciplinary teams, or participation in clinical trials, and who may therefore experience different outcomes driven by structural barriers to care rather than biological differences. In a large real-world analysis conducted by Biondo et al., Hispanic/Latino patients, who comprised 8% of the study population, demonstrated OS and time to second-line therapy or death outcomes comparable to those of non-Hispanic White patients after adjustment for baseline characteristics. In this report, although Hispanic/Latino patients were younger and more likely to have Medicaid insurance, race/ethnicity itself was not significantly associated with inferior survival outcomes [38].

## 5. Conclusions

Our findings support the concept that age and comorbidities should not limit therapy with curative intent. Comorbidities and reduced functional status may necessitate dose reduction or medication adjustments, as well as supportive care interventions.

This study demonstrates that R-mini-CHOP achieved response and survival outcomes comparable to those of R-CHOP in elderly patients with DLBCL or high-grade follicular lymphoma despite a higher age and comorbidity burden.

We observed a trend toward higher ORR in Hispanic patients treated with R-CHOP, although this difference was not statistically significant. We did not observe survival differences by ethnicity; however, this observation may be confounded by selection bias and should be validated in larger datasets. In summary, reduced-intensity R-mini-CHOP produced comparable long-term overall survival to R-CHOP in elderly patients despite worse baseline characteristics.

Treatment regimens incorporating novel agents are delivering encouraging outcomes for elderly Hispanic patients in clinical trials. Prospective multicenter cohorts are essential to confirm these results and to define effective treatment strategies for aging and ethnically diverse populations, as this demographic is expanding faster than anticipated.

Together, the OS and PFS curves support the conclusion that R-mini-CHOP is an effective and well-tolerated alternative to R-CHOP in elderly patients with aggressive B-cell lymphomas, even in those with advanced age, comorbidities, and higher-risk features. The absence of significant differences in survival outcomes reinforces the clinical utility of R-mini-CHOP for frail or very elderly populations, especially in real-world settings in a predominantly Hispanic cohort of patients. The lack of significant survival differences by ethnicity in our cohort may reflect equitable access to specialized care, contrasting with historical disparities observed in aggressive lymphoma outcomes among minority populations.

## Figures and Tables

**Table 1:** Patient characteristics.

	R-mini-CHOP *N* = 72 (%)	R-CHOP *N* = 64 (%)	Effect Size (Odds Ratio)	*p*-Value
Age	82 [70–94]	74 [70–83]	−7 (−3 to −9)	**<0.001**
70–80 years	21 (29%)	9 (14%)	Ref	**<0.001**
>80 years	51 (71%)	55 (86%)	14.841 (6.225 to 35.385)	
Sex				
Male	30 (42%)	29 (45%)	Ref	0.669
Female	42 (58%)	35 (55%)	1.160 (0.588 to 2.289)	
Race				
White	61 (85%)	54 (84%)	Ref	0.925
Other	8 (11%)	8 (13%)	0.885 (0.311 to 2.520)	
Unknown	3 (4%)	2 (3%)	1.328 (0.214 to 8.247)	
Ethnicity				
Hispanic	42 (58%)	31 (48%)	1.490 (0.756 to 2.936)	0.249
Non-Hispanic	30 (42%)	33 (52%)	Ref	
Diagnosis				
DLBCL	62 (86%)	57 (89%)	Ref	0.604
High-grade FL	10 (14%)	7 (11%)	1.313 (0.469 to 3.681)	
Ann Arbor III-IV				
Yes	53 (74%)	40 (63%)	1.674 (0.808 to 3.468)	0.166
No	19 (26%)	24 (38%)	Ref	
High LDH				
Yes	31 (43%)	30 (48%)	0.832 (0.421 to 1.641)	0.595
No	41 (57%)	33 (52%)	Ref	
EN > 1 site				
Yes	12 (17%)	17 (27%)	0.553 (0.241 to 1.270)	0.163
No	60 (83%)	47 (73%)	Ref	
High-Risk IPI				
Yes	22/60 (37%)	10/58 (17%)	2.779 (1.176 to 6.568)	**0.020**
No	38/60 (63%)	38/58 (63%)	Ref	
High-Risk NCCN-IPI				
Yes	22 (31%)	4 (6%)	6.600 (2.133 to 20.422)	**0.001**
No	50 (69%)	60 (94%)	Ref	
Double Expressor				
Yes	35/57 (61%)	21/50 (42%)	2.197 (1.013 to 4.766)	**0.046**
No	22/57 (39%)	29/50 (58%)	Ref	

Abbreviations: DLBCL: diffuse large B-cell lymphoma; FL: follicular lymphoma; LDH: lactate dehydrogenase; EN: extranodal; IPI: international prognostic index. Bold values indicate statistical significance (*p* < 0.05).

## Data Availability

The raw data supporting the conclusions of this article are not publicly available due to ethical and privacy restrictions, as they contain protected health information (PHI). A limited, de-identified dataset may be made available by the corresponding author upon reasonable request. Requests will be reviewed to ensure they are for legitimate scientific inquiry and will require the completion of a data use agreement (DUA) in accordance with institutional policies.

## Abbreviations

The following abbreviations are used in this manuscript:

DLBCL	Diffuse large B-cell lymphoma
FL	Follicular lymphoma
OS	Overall survival
PFS	Progression-free survival
LDH	Lactate dehydrogenase
SEER	Surveillance, Epidemiology, and End Results
WHO	World Health Organization
ECOG	Eastern Cooperative Oncology Group
IPI	International prognostic index
FISH	Fluorescence in situ hybridization
EMR	Electronic medical records
IRB	Institutional Review Board
ORR	Overall response rate
EFS	Event-free survival
CR	Complete response
PR	Partial response
CGA	Comprehensive geriatric assessment
ABC	Activated B-cell
GCB	Germinal center B-cell
GELA	Groupe d’Etude Des Lymphomes de l’adulte
Chemotherapy Regimens:	
R-CHOP	Rituximab, cyclophosphamide, doxorubicin, vincristine, and prednisolone
R-mini-CHOP	Dose-attenuated R-CHOP
R2CHOP-21	Lenalidomide plus R-CHOP
R-COMP	R-CHOP without doxorubicin
R-miniCEOP	R-CHOP with epirubicin replacing doxorubicin
RCHOP-14	R-CHOP administered every 14 days
Pola-R-CHP	Polatuzumab vedotin, rituximab, cyclophosphamide, doxorubicin, and prednisone
Pola-R-miniCHP	Dose-attenuated Pola-R-CHP

## Appendix A

**Table A1:** Predictors of overall survival.

Univariate Analysis	Hazard Ratio	95% CI	*p*-Value
Sex			
Female	0.676	0.347–1.315	0.248
Age	1.043	0.984–1.105	0.160
Ann Arbor			
III-IV	1.254	0.602–2.614	0.545
Hispanic	0.924	0.476–1.794	0.815
High-risk IPI	1.439	0.658–3.149	0.362
NCCN-IPI	2.261	1.053–4.855	**0.036**
High LDH	3.062	1.520–6.169	**0.002**
Treatment			
R-CHOP	Ref		
R-mini-CHOP	1.012	0.517–1.983	0.972

Abbreviations: IPI: international prognostic index; LDH: lactate dehydrogenase. Bold values indicate statistical significance (*p* < 0.05).

**Table A2:** Predictor of response—univariate analysis (response= CR).

Univariate Analysis	Odds Ratio	95% CI	*p*-Value
Sex			
Female	0.868	0.298–2.531	0.796
Age (continuous)	0.940	0.853–1.036	0.215
Ann Arbor			
III-IV	0.425	0.113–1.604	0.207
Hispanic	0.868	0.298–2.531	0.796
High-risk IPI	0.387	0.120–1.247	0.112
NCCN-IPI	0.272	0.078–0.943	**0.040**
High LDH	0.158	0.047–0.534	**0.003**
Treatment			
R-CHOP	Ref		
R-mini-CHOP	0.728	0.250–2.123	0.561

Abbreviations: IPI: international prognostic index; LDH: lactate dehydrogenase. Bold values indicate statistical significance (*p* < 0.05).

**Table A3:** Hispanics predictors of survival.

Univariate Analysis	Hazard Ratio	95% CI	*p*-Value
Sex			
Female	0.790	0.304–2.053	0.629
Age	1.070	0.982–1.167	0.124
Ann Arbor			
III-IV	1.176	0.413–3.352	0.761
High-risk IPI	1.887	0.633–5.618	0.254
NCCN-IPI	3.068	1.038–9.036	**0.043**
High LDH	3.616	1.321–9.901	**0.012**
Treatment			
R-CHOP	Ref		
R-mini-CHOP	1.721	0.640–4.629	0.282

Abbreviations: IPI: international prognostic index; LDH: lactate dehydrogenase. Bold values indicate statistical significance (*p* < 0.05).
